# Manure management during African swine fever outbreak: identifying gaps through a narrative review

**DOI:** 10.3389/fvets.2026.1760996

**Published:** 2026-05-13

**Authors:** Anna Macdonald, Ngo Omoaka, Manuel Juarez, Javier Bahamon, Emma Stephens, Roland Kroebel, Claudia Narvaez, Aruna Ambagala, Philip O. Soladoye

**Affiliations:** 1 National Centre for Foreign Animal Disease, Canadian Food Inspection Agency, Winnipeg, MB, Canada; 2 Faculty of Agricultural and Food Sciences, University of Manitoba, Winnipeg, MB, Canada; 3 Agriculture and Agri-Food Canada, Lacombe Research and Development Centre, Lacombe, AB, Canada; 4 Alberta Pork, Quality Assurance and Production, Edmonton, AB, Canada; 5 Agriculture and Agri-Food, Lethbridge Research and Development Centre, Lethbridge, AB, Canada

**Keywords:** African swine fever, biosecurity measures, disease control, environment, manure management, proactive approach

## Abstract

African swine fever (ASF) is a significant threat to the pork industry, a sector vital to food security and the agro-economy within Canada and across the globe. While ASF has not yet been introduced to Canada, its recent detection in the Americas highlights the urgent need for preparedness and effective response strategies to mitigate viral transmission in the event of its incursion on Canadian soil. Appropriate disinfection of contaminated materials is crucial to strengthening biosecurity measures. In particular, manure could serve as a reservoir for the virus, and appropriate treatment protocol must be established to eliminate manure as a source of ASF spread. However, literature to inform effective corrective actions are sparse and inconsistent. The goal of this narrative review is to provide readers with a basic understanding of ASF virus (ASFV), socioeconomic and environmental impacts of ASF, common manure management practices, and how manure treatment practices can promote or hinder biosecurity in the event of an ASF outbreak. This paper also compiles relevant publications to begin a necessary discussion on safe treatment methods and containment strategies to reduce the spread of the virus, as well as to identify and bridge critical gaps between ASFV and manure management to inform the development of effective manure management protocols, thereby enhancing Canada's preparedness for a potential ASF outbreak.

## 1 Introduction

Pork production is a key agricultural sector in Canada, significantly contributing to the national economy and employing 103,000 persons across the country (1). The Canadian pork industry is predominantly focused on export with approximately 70% of its products sold internationally. Canada is the third largest pork exporter globally by value and volume (1). In 2020, 1.5 out of 2.3 million tons of pork were exported around the globe, with the USA, Japan, and China being top markets (2). In addition to impressive production capacity, the industry also adapts readily to fluctuating market pressures. Following widespread closures of American processing plants during the COVID-19 pandemic and a coinciding ASF outbreak in China, Canadian producers increased slaughter volume between 2019 and 2020 by 4% to address shortages, highlighting the resilient nature of the industry (3).

**Figure 1:**
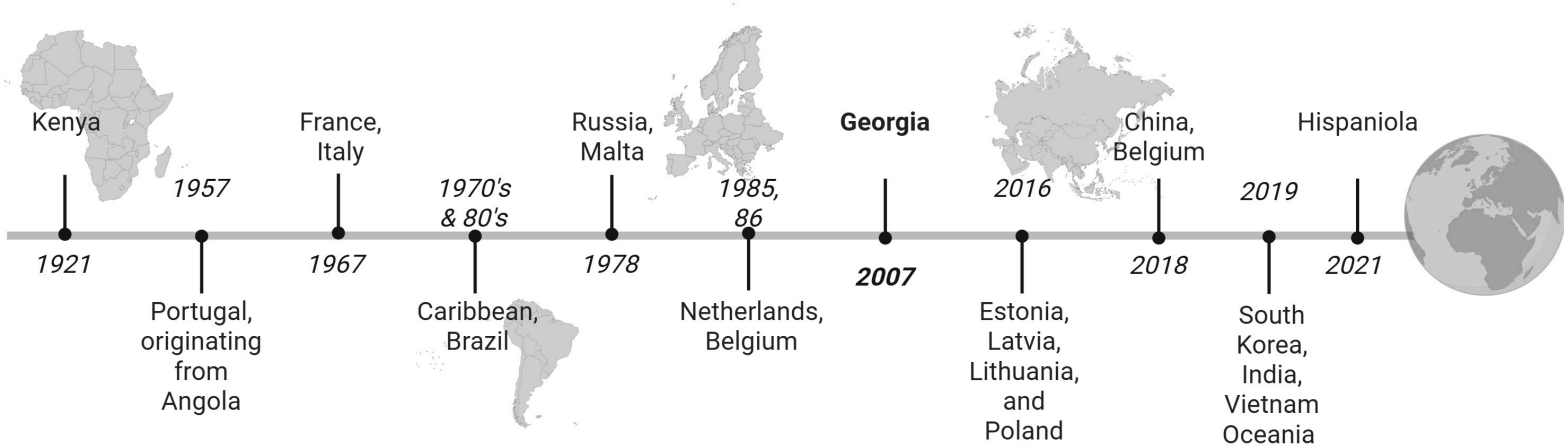
Timeline of ASFV spread across the globe, highlighting key jumps between geographic regions. Introduction of highly virulent, genotype II into continental Europe represented in bold (Generated with BioRender). © His Majesty the King in Right of Canada, as represented by the Minister of Agriculture and Agri-Food.

ASF continues to spread globally creating a persistent, high-mortality crisis for the swine industry with profound economic and ecological consequence (Figure 1). It is a contagious viral disease caused by ASFV that affects both domestic and wild Eurasian pigs (4). ASFV has been circulating in the sylvatic cycle between soft ticks and wild pigs (warthogs, bush pigs and giant forest pigs) in eastern and southern Africa for decades. The first intercontinental spread of ASFV occurred in 1957 from Sub-Saharan Africa to Portugal through human-mediated transport of infected pork (5). Subsequently, the virus (highly virulent genotype I) spread throughout Europe, to the Caribbean and Brazil (6, 7). Widespread campaigns to cull and disinfect contaminated farms stemmed the magnitude and frequency of outbreaks, with many countries self-declaring ASF free status in the late 20th century. In 2007, Georgia reported detection of a highly virulent genotype II virus (5). Subsequently, the virus spread throughout the Caucasus region and since then, ASF has periodically resurfaced throughout Europe, and spread to Asia, Oceania, and the Island of Hispaniola (8, 9, 162) (Table 1). With the absence of a globally accepted vaccine, the virus is expected to persist and potentially increase in scope, driven by its high resilience in the environment, complex transmission pathways, the effects of climate change, and income disparities (5, 10–12).

**Table 1:** Chronological Progression of Spread of ASF to regions previously free from pathogen.

Year	Country	Reference
1957; 1960–1993; 1999	Portugal	(129)
1964, 1967, 1977	France	(130)
1967, 1980	Italy	(130)
1971, 1981	Cuba	(7)
1977	Russia	(131)
1978	Malta	(132)
1978–1980	Dominican Republic	(133)
1978–1984	Haiti	(133)
1978–1981	Brazil	(6)
1985	Belgium	(134)
1986	Netherlands	(135)
2007, 2010, 2011	Georgia	(136)
2007–2014	Armenia	(8)
2007	Russia	(136)
2008	Azerbaijan	(8)
2008	Iran	(137)
2013	Belarus	(8)
2014	Estonia, Latvia, Lithuania, Poland	(138–141)
2016	Moldova, Czech Republic, Romania, Bulgaria, Hungary	(141, 142)
2017	Georgia, Armenia, Azerbaijan, Russia, Belarus	(8)
2018	Southern Belgium, China	(143)
2019	Slovakia, Serbia, Cambodia, Greece, East Timor, Myanmar, South Korea, Indonesia, Vietnam, Philippines	(8, 144, 145)
2020	Papau New Guinea, India	(8)
2021	Dominican Republic and Haiti, North Macedonia, Thailand	(8)
2022	Italy, Nepal	(8)
2023	Singapore, Bosnia and Herzegovina, Croatia, Sweden, and Bangladesh	(8)
2024	Montenegro and Albania, Bhutan, Cote D'Ivoire, Germany, Poland, Sri Lanka, Latvia, Ukraine	(8)
2025	Malaysia, Cabo verde, Romania	(8)

ASF is a reportable disease in Canada (163), a notifiable disease in the European Union (164), and must be immediately reported to the World Organization for Animal Health (165). Early detection of ASF is however challenging due to the complex clinical presentation of the disease that mimics other common swine diseases, and due to logistical difficulties in conducting extensive surveillance in both domestic and wild suid populations. The disease often presents with high fever, respiratory distress, and sudden death, leaving little time for diagnosis (13) (Table 2).

**Table 2:** Severity of different forms of ASF and influence on shedding in excretions (18, 19, 146–148).

Clinical form	Symptoms	Likeliness of ASFV Shedding in Manure
Peracute	High fever, erythema, no seroconversion, rapid death	Low
Acute	Fever, reduced appetite, lethargy, cyanosis, vomiting, diarrhea, respiratory distress, hemorrhages in the skin and internal organs, spontaneous abortion, high mortality (up to 100%).	High
Subacute	Intermittent fever, reduced appetite, lethargy, low mortality (30%−70%)	Moderate
Chronic	Lingering clinical signs including weight loss, respiratory issues, skin necrosis, chronic ulcers, and swollen joints	Moderate

As stipulated by the World Organization of Animal Health (WOAH), when an animal tests positive for ASF, the affected country loses ASF free status and is subjected to export restrictions until no positive cases have been detected for three consecutive years (14). Moreover, other countries may be inclined to impose additional trade restrictions (15). Therefore, to minimize the impact of ASF on the pork industry and to restore operations in a timely manner, a rapid outbreak response is imperative. With no available therapeutic treatments, herds must be immediately depopulated, and their waste and farm infrastructure must be decontaminated promptly to control the spread of the disease.

Since manure is difficult to adequately disinfect, swine excrement can promote environmental ASFV persistence, increasing the chance of lengthy outbreaks. ASFV infected pigs shed the virus in all bodily fluids and excretions, including urine, feces, and oronasal secretions, even before the onset of clinical signs (16). The amount of virus shed by the ASFV infected pigs is influenced mainly by the virulence of the virus (Table 2) (17, 18). Pigs infected with low virulent ASFV strains continue to shed the virus in secretions and excretions for several weeks (17). Because of high viral loads in blood, when the pigs develop bloody diarrhea during advanced stages of the disease, manure can be heavily contaminated with ASFV (16, 17, 19). Manure serves as a protective matrix, by shielding virus particles (physical barrier) from exposure to chemical disinfectants and environmental factors, and also by quenching the disinfectants before they reach the virus (20). Contaminated manure generated in large volumes could serve as a source of infection for susceptible pigs when held on site. Additionally, manure can exacerbate spread of the virus to surrounding farms when transported and applied as fertilizer or carried on boots and transport trucks (21). Therefore, manure from ASF infected farms could be a major, and often underestimated, source of indirect transmission. The ability of ASFV contaminated manure to promote disease transmission to susceptible pigs has been proven experimentally (22), particularly if exposure to contaminated materials is repeated (10). While several studies have assessed disinfection of ASFV under laboratory conditions (20, 23–29), practical and effective methods to decontaminate ASF-contaminated manure, while minimizing economic losses and environmental impact, have not been established, highlighting a critical risk for the Canadian agricultural sector.

The goal of this narrative review was to compile available information from academic and non-academic sources on swine manure management practices and factors affecting manure treatment to provide a single, consolidated resource to familiarize readers who may lack a comprehensive and complete understanding of the intricacies involved in managing manure following an animal disease outbreak. Initially, search queries specifically focused on treatment of ASFV in swine manure, but due to the sparsity of relevant publications, an iterative search approach was adopted to review ASFV and manure management independently. Scopus, PubMed, and Google databases were used to find literature which provided information on ASF, swine waste management, or ideally, an overlap of these two topics. Publications were excluded if they focused heavily on non-swine manure, as these systems are less relevant when concentrating on a disease that affects only swine species. Additionally, since our research focused on ASFV, a virus with unique physiology, resources were typically excluded if they focused heavily on other virological agents, unless they provided some relevant context. By compiling and weighing the related literature, we synthesized a discussion on potential strategies for ASF manure management and identified the numerous gaps in scientific study which must be addressed. This review bridges findings on ASFV and manure management, providing a foundation for developing robust manure management strategies and response preparedness in the event of ASF incursion into Canada.

## 2 African swine fever virus

ASFV is a large morphologically and genetically complex, double-stranded DNA virus with a large genome (170–193 kilobase pairs) encoding viral proteins known to promote virulence, immune evasions, and host cell process modulation (4, 30). ASFV is unique in many aspects, separating it from other animal viruses. It is the sole member of the *Asfaviridae* family, and the only DNA arthropod borne virus known to date (31, 32) ASFV is characterized by a sophisticated five-layered structure consisting of an outer envelope, capsid, inner envelope, core shell, and nucleoid essential for its exceptional stability and resistance to environmental degradation (33). The viral particle is stable under a broad range of environmental conditions, allowing it to persist in inhospitable settings, particularly in colder temperatures (34). ASFV can survive several freeze thaw cycles (35) and is highly resistant to pH changes. It can maintain infectivity across a wide range of pH (4–13), and its tenacity is enhanced when in organic materials (34). ASFV can survive a temperature of 56 °C for over an hour. Inactivation time for ASFV in swill, is 119 min at 70 °C and 4 min at 96 °C (36). In slurry, ASFV is highly resistant to heat compared to other high consequence RNA viruses such as foot and mouth disease virus, classical swine fever virus, bovine viral diarrhea virus, and swine influenza virus, and swine influenza viruses (37). These findings have also been supported by other studies comparing DNA and RNA virus persistence in human excreta and animal manure (38). However, Turner and Williams (24) noted that ASFV is less resilient compared to swine vesicular disease virus, likely due to the enveloped nature of ASFV. Thus, additional studies on ASFV survival in manure are critical to develop protocols to manage manure generated in ASF infected pig farms. Generalizations based on other viruses may lead to misleading conclusions.

## 3 Socioeconomic impact of ASF

An outbreak of ASF can have devastating social and economic implications on affected regions due to loss of livelihood, necessary depopulation events, and other costs incurred during an outbreak (39). Indirect factors, such as reduced consumer trust, herd restructuring, and shifts in investment patterns, further the negative impacts on the pork producing sector and those it employs (40, 41). Globally, swine farming is an integral part of life for many groups, serving as a staple protein source, indication of social status, and component of cultural identity. In many developing regions, farming is relied upon to provide a stable means of income, a reliable source of protein, or as an extra resource which can be sold in the event of financial hardship (5, 39, 42). For small-holder farms in endemic countries, an ASF outbreak can equate to loss of occupation, their sense of identity, and economic stability, which can have trickle down effects on financing for their children's education and healthcare costs and lead to long term deficits for the family (43). The negative effects are also felt by those peripherally involved with the farm through feed production, veterinary services, transport, and meat processing industries (43).

The economic ramifications of ASF outbreaks can be quantified either by retrospectively analyzing the costs incurred during an outbreak, or by using predictive modeling to estimate costs in a fictional, but possible, scenario. Various economic indicators can be used to assess the magnitude of former outbreaks around the globe, including compensation paid to farmers, cost per head of swine culled, or reduction of market value. In Haiti during ASFV genotype I outbreak (1981–1984), 384,391 swine were culled, resulting in compensation of over USD $9 million paid to farmers (9). Babalobi et al. (44) reported financial costs incurred in the Oyo State in Nigeria, where nearly 30,000 pigs were culled in 2001, resulting in a loss of USD $941,492. Outbreaks in Benin in 2014-2018 and Tanzania in 2019 lead to losses of USD $1,587,174 and USD $41,065, respectively (45, 46).

After the recent introduction of ASF into Asia, total losses in China were approximately USD $111 billion, demonstrating an extreme scenario in which the largest pork producing nation faced severe losses (47). Vietnam experienced ASF outbreaks after China, where approximately 20% of the nation's pig population was culled, leading to losses estimated to be between USD $880 million to 4.4 billion (48), as was the case in India, where 54,150 pigs were culled, equalling approximately USD $37.23 million in losses (39). Additionally, indirect costs arising from disposal of contaminated materials and carcasses, purchasing disinfectants, and diagnostic testing increased the total cost of the outbreak in India. A 2019 outbreak in the Philippines led to a loss of approximately $58 million (40).

Depending on the overall population, disease control strategies, surveillance, market flexibility, and local prices (labour, materials, animals, etc.), costs of an outbreak can fluctuate greatly between countries, as reported by Casal et al. (40). For example, costs would vary drastically between a developed country like Canada, compared to a developing nation with predominantly subsistence, household farms. Modeling by Casal et al. (40) analyzed a hypothetical scenario in which an outbreak affecting 18 farms in North Macedonia could cost over USD $6 million. Biden et al. (15) designed a model to estimate various scenarios in the Canadian market, examining repercussions of outbreaks arising in Quebec, Ontario, or Western Canada. Owing to differences in export volumes between the different regions, the three scenarios would have varying impacts on the pork industry, with economic losses between CAD $3.59–3.76 billion if the outbreak affects operations for 24 months. A report by Slatyer et al. (49) explored the potential consequences of ASF introduction to domestic or feral hogs in Australia, noting estimates of AUD $101–263 million to successfully eradicate the virus. Moreover, the cost associated with ASF becoming endemic to Australian swine populations could amount to AUD $2.5 billion.

Bech-Nielsen et al. (50) weighed the benefits and costs of eradication of ASF from Spain, demonstrating that investments to eradicate the disease, while costly, will avoid losses in the long-run. While some developed nations invest more heavily in preventative measures, those with less economic means have less protection during an outbreak, and ultimately, incur more losses in the long term. Looking ahead, it will be important for Canada to consider ways to continue strategic investment to proactively prepare response strategies to minimize the losses if outbreaks were to occur. Funding from the Government of Canada has been invested toward biosecurity assessments, improvements to abattoirs, wild pig management, and sector analysis through the ASF Industry Preparedness Program. Additionally, the Canadian government is actively reviewing best approaches to culling and disposal, and development of the Pan-Canadian ASF Action plan (51). Continuing efforts are aimed to prevent ASF from entering Canadian farms and to further bolster preparedness strategies in the event that the disease is potentially detected.

While economic estimates serve as a tangible measure of the scale of an ASF outbreak, the effects of a foreign animal disease outbreak can have more covert consequences for those involved in animal disease outbreak response. A review of the psychological impacts of a foot and mouth disease outbreak in the UK in 2001 discussed the human health outcomes of livestock depopulation events (52). Farmers may experience significant stress, anxiety, and depression due to financial losses, job insecurity, and the trauma of culling animals. These outcomes were similarly documented by Purc-Stephenson and Doctor (53) in the Canadian context for animal diseases, such as FMD in swine operations and avian influenza in poultry flocks. Farmers described emotional distress, shock, and anxiety following depopulation of their animals, adding feelings of loss of identity, economic worries, and distrust for governing agencies. Moreover, the public may experience negative emotions when observing large piles of deadstock or carcass burning, anxieties of a disrupted food supply, or concerns of zoonotic spillover. The negative public opinions of animal culling were highlighted recently in British Columbia, Canada, when a flock of ostriches was culled following detection of highly pathogenic avian influenza. After lengthy legal battles between the farm and the Canadian Food Inspection Agency, the depopulation of the flock was widely publicized, with the public noting feelings of distrust in governmental agencies and concerns over animal welfare (54). Efforts are needed to anticipate and mitigate adverse health outcomes which may be experienced by farmers, other affected personnel, and the public. While the focus of animal disease outbreaks predominantly centers on the loss of quality of animal health, there are significant emotional and psychological impacts borne by those involved in addressing the outbreak, and the entirety of the combined socioeconomic effects must be considered to appreciate the gravity of infectious disease outbreaks.

## 4 Environmental impact of ASF

Broadly, animal disease outbreaks can have major impacts on environmental health by accelerating habitat degradation, increasing the input of water required in a farm operation (i.e., for cleaning and disinfection purposes), increasing the risks of biological or chemical contamination, and elevating greenhouse gas emissions (55). During an ASF outbreak, many animals need to be culled, resulting in scores of infected carcasses which require proper disposal. Depending on resources available, carcasses may be disposed of through burning, burial, or composting, each of which carry environmental consequences. Incineration, while effective in destroying the virus, can release airborne pollutants, such as dioxins and furans, and is impractical for areas with arid conditions susceptible to forest fires (56–58). Deep burial is unsuitable for areas with high groundwater levels or proximity to water sources, as it can lead to water contamination (56). Moreover, deep burial is generally accompanied by application of lime to accelerate pathogen inactivation, increasing the risk of chemical hazards introduced into the environment.

In addition to carcass disposal, materials and manure generated in an outbreak need to be thoroughly disinfected to eliminate infectious viruses. Cleaning and disinfection requires the input of freshwater and chemical sanitizers, the latter of which (including aldehydes, sodium hydroxide, lime, iodine, and phenols), are known to have toxic effects on the environment (59). On its own, swine manure can lead to environmental issues, such as nutrient runoff contaminating water systems, air pollution from ammonia and greenhouse gas emissions, soil degradation from nutrient imbalances, and heavy metal accumulation (60, 61), all of which must be addressed throughout outbreaks, when potentially large amounts of manure requiring processing while operations are complicated and stressed by strict quarantine measures. Contaminated manure pits or lagoons must be strictly contained to prevent risk of runoff and eutrophication, particularly in the event of high precipitation (62).

## 5 Manure management

**Figure 2:**
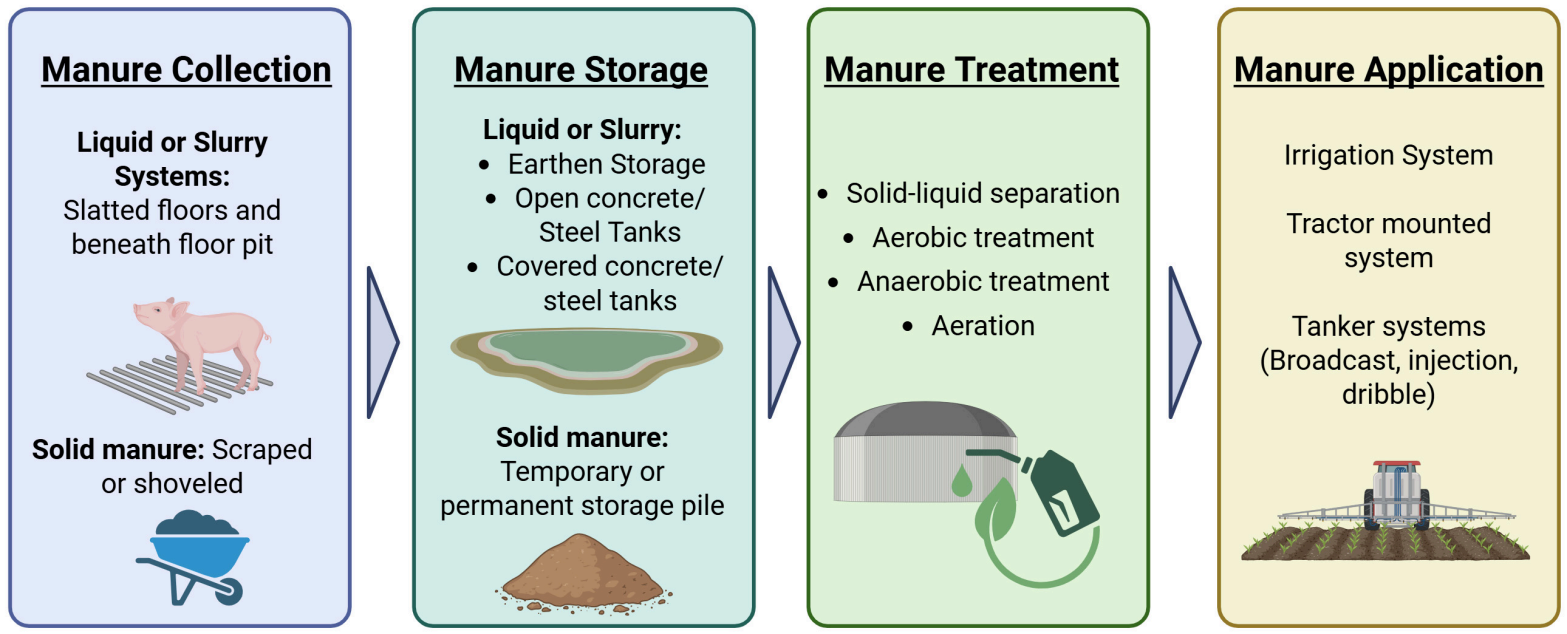
Overview of key manure management processes. Generated with BioRender.

While often overlooked or viewed as a byproduct of farming, livestock manure is a resource critical for fertile soils and abundant crop production. Successful management of manure is essential for any livestock farm, comprising collection, storage, treatment, and application of manure (63) (Figure 2). Manure is a complex mixture of fecal material, undigested feed, urine, bedding, and wastewater (64), the composition of which varies based on characteristics unique to each farm, including livestock age, breed, nutrition, and housing, ranging greatly in physical, biological, and chemical composition (65). Manure management strategies may apply across different regions depending on their regulatory framework and the specific characteristics of the location such as soil type, proximity to waterways, geology, and depth of groundwater (65, 66). Globally, approaches vary drastically, depending on climate, local regulations, herd size, and economic investment. This section reviews the general processes of manure management. Since economic resources largely dictate strategies undertaken, the discussion focuses on the approaches of developed and developing systems, highlighting factors which can compromise or enhance biosecurity. A review of styles and stages of manure management provides a working foundation for discussing feasible manure treatment strategies for ASF in Section 6.

### 5.1 Manure management in developed systems

In developed regions, farming systems have largely evolved into efficient, industrialized models which provide consumers with a stable market of consistently affordable pork. The integration of the hog supply chain allows producers to streamline production systems, invest in advanced infrastructure, access superior feed, and reliable veterinary services (67, 68). Manure management is similarly optimized and properly appreciated in highly integrated models. Within North America, livestock manure regulations are dictated by either state or provincial government and vary according to the region (69), but broadly speaking, manure management aims to promote environmental sustainability, animal health, and farm profitability (70). Across developed systems, such as North America, Europe, and Australia, manure management reflects similar approaches, with larger, consolidated farms geared toward optimizing production while adapting techniques to minimize greenhouse gas emissions and enhance nutrient optimization (66, 71–73). For example, the average swine farm in Canada houses approximately 1850 pigs in 2021 compared to 920 in 2001, demonstrating the trend toward larger, commercial sized farms (74).

#### 5.1.1 Manure collection

The first stage of manure management is the collection of manure generated by the pigs. The method depends on how the pigs are housed (pasture, dry lot, or enclosed barn) and growth stage (gestating, farrowing, nursery, or finishing) (64). Waste can be collected as solid (>20% solid content), semi-solid or slurry (5%−20% solid content), or liquid manure (< 5% solid content) (75, 76). Solid systems are utilized for certain styles of pig-rearing, such as smaller farms where pigs forage and manure can be incorporated directly into local soil (77). Farms using hoop structures (large, tarped, cylindrical units which are affordable and well-ventilated) often collect solid manure and incorporate straw bedding into the solid waste (75). Solid manure is typically scraped, shoveled, or hauled into piles for storage, representing a low investment strategy which can be easily maintained on smaller operations (78).

However, on the majority of modern operations, manure collection tends toward liquid/slurry storage systems which rely on gravity to enable efficient collection of manure beneath slatted floors (72). In liquid collection systems, pigs are moved between areas of the barn, and waste is flushed with freshwater downward through slats into holding pits (70). The barn interior can be easily cleaned and disinfected properly in between cohorts of animals. Additionally, these systems limit the contact of animals and pathogen-containing excretions compared to solid systems, which house animals more closely with the manure they generate. The frequency of emptying the collecting pits can vary depending on the mechanical systems and depth of the pits, with scraper and flush gutter systems emptied every 1–2 days, whereas deep-pits and pull-plug systems are emptied less frequently, between 2 weeks to several months (72). Liquid or slurry manure is then pumped from pits beneath the barn into holding pits, tanks, or lagoons (77). Europe, the United States, and Australia largely employ liquid/slurry systems to optimize nutrient recovery while managing large herd sizes (66, 71, 72). In Canada, 92% of swine operations reported using a liquid collection system (79). Overall, liquid systems are practical for intensive operations, facilitating efficient cleaning and distribution of manure to nearby cropland, whereas solid systems are cost-efficient and practical for farms with less extensive infrastructure.

#### 5.1.2 Manure storage

As the average hog farm has grown in size, the volume of manure produced has increased and surpassed the capacity of direct application onto cropland (68). Now, there is a greater need to store manure, short-or long-term, in a safe and sustainable manner (80). Various structures are employed to effectively contain the manure, and again, are largely dependent on whether manure collected is liquid/slurry or solid. Built implements include collection pits beneath the barn, a gravity transfer pump into a large storage tank, and eventually, large lagoons or tanks with the capacity to store manure until treatment or application (75). Liquid manure can be stored in-building or outdoors in concrete, steel, or earthen tanks, pits, basins, or lagoons, which may be lined or unlined, covered or uncovered, and above or below ground, depending on the farmers' needs (79). Depending on depth and aeration, lagoons and holding ponds will range between aerobic, facultative, or anaerobic conditions. As many developed countries are shifting toward methods with reduced environmental impact, lagoons are commonly covered to decrease odor and reduce methane emissions (72, 81). Lagoons may either be aerated or left to fallow to favor aerobic or anaerobic digestion, respectively (64). Over time, manure solids accumulate in a bottom layer of sludge, whereas the liquid manure collects on top, and is emptied more frequently. Lagoons require proper maintenance to ensure the contents are being effectively managed and meet operating requirements.

Solid manure can be stored in either temporary or permanent sites (66, 70). Temporary storage sites are kept on the field and used directly for crop production (65). While the strategy is efficient, storage must be short term, located a safe distance from a watercourse, and on specific soil conditions (i.e.; slope < 3%, on select soil types, etc.) to avoid contaminating ground or surface water (70). For permanent storage, a durable concrete or steel surface should be included under the manure pile, and consideration should be paid to the soil supporting the storage pad, side walls to limit surface water flow and facilitate easy collection with a front-end loader, and inclusion of a roof to limit the role of precipitation (65, 77, 82).

Other storage systems allow for solid/liquid separation in multi-tank systems in which gravity separates the solid fraction from the liquid portion in primary and secondary storage cells (83). Such systems effectively partition solids from liquids, which can then be directed to different uses. Regardless of the type of manure storage, protecting the surrounding environment is critical to ensuring a safe and sustainable manure strategy. Storage must incorporate controls to limit odor, environmental impacts, safety issues, and promote efficiency for the workers on the farm, while adhering to local legislation (65, 77).

#### 5.1.3 Manure treatment

Manure treatment alters the inherent physical, chemical, and biological properties to facilitate easier storage, application, or disposal (66). Treatment can modify manure moisture content to reduce volume, simplify hauling, create a value-added product, minimize nutrient loss, or limit environmental risks (65). While many farms can apply manure without treatment, treatment strategies can optimize surplus manure or tailor it to specific farming needs. Common manure treatment strategies include anaerobic digestion, aerobic treatment, solid/liquid separation, and aeration (66).

Anaerobic digestion is a naturally occurring process by which microorganisms degrade organic material in the absence of oxygen and allows for capture of methane from manure which can be used as a renewable energy source. Anaerobic biodigesters operate under controlled conditions which promote the biochemical reactions of anaerobe communities, between 15 °C and 45 °C and pH of 6.5–7.5 (70). Digesters can range from simply covering lagoons to accelerate methane production, to sophisticated tanks systems with engineered capture mechanisms (72). Liquid or solid digestate can then be used to form value-added products, such as fertilizer, bioplastics, horticultural products, animal bedding, or to reuse water (70, 84). Additionally, biogas rich in methane and carbon dioxide can be used as an alternative to natural gas to heat homes and generate electricity (81). Anaerobic digestion reduces odor and pathogens, and provides an alternative to fossil fuels, elevating its popularity (70). However, the development of a modernized digestion plant can be costly and require expertise, high labor inputs, and constant maintenance (85). Moreover, improperly implemented digestor systems can also be problematic. With heavy focus on methane gas recovery from biodigesters, the nutrient-rich digestate may be neglected and treated as an afterthought, lacking integration into croplands (63). The strategy is becoming widely adopted across sectors which have sufficient investment to implement the necessary infrastructure, gaining traction in Brazil, Argentina, the United States, Canada, Australia, and throughout the European Union (66, 71, 85, 86). Interestingly, China also relies on large-scale anaerobic digesting. The Chinese pork sector has been greatly industrialized to meet consumer demand, which is reflected in their manure management (87). The “Gan qing fen” technique is used to collect manure and is mandated in the Beijing area (87). Feces collected from concrete floors are composted and applied to fields, whereas liquid wastes are treated in biogas plants, which produces fuel for local villages. Biogas plants provide an outlet for the excess manure, transforming it into valuable cooking fuel. Although considered to be developing regions, China, Brazil, and Argentina are unique examples of how investments in modernized infrastructure can shape farming operations and practices, and as a result, influence biosecurity measures.

Aerobic digestion is an alternative method used for solid manure which provides the bacterial population with oxygen and a carbon source (65). Composting transforms solid manure into stable products which can be applied as fertilizer. Conditions should be optimal for the microbiota present, including a carbon to nitrogen ratio between 20:1 and 30:1, oxygen content >10%, pH between 6.5 and 8 and temperature between 55 °C and 60 °C (65). Composting is suitable for material with moisture between 40% and 65%, as high moisture content may disrupt the aerobic process. Hence, solid/liquid separation may be required for liquid/slurry manure. Composting reduces manure volume, pathogens and weeds, and generates a nutrient-rich product which can be sold or spread.

Solid/liquid separation divides liquid or slurry manure into a solid and liquid fraction, generating two products with different applications. Various techniques can be used to separate the components, including screw presses, belts, screens, flocculation, and coagulation. While the strategy provides two products with improved properties (a solid fraction with highly concentrated nutrient that is easy to store and transport and a liquid fraction that can be pumped and applied efficiently), it does rely on a more complicated mechanism which increases equipment, maintenance, and labor costs (70). Aeration treatment uses heat to remove moisture from liquid manure, facilitating packaging, and hauling (76). Additionally, manure additives are compounds applied to manure piles with the goal to reduce unfavorable characteristics of manure, including odor and gaseous emissions. While there is debate on whether the products are significantly effective, but their adoption may grow as new products are developed and research supports their inclusion (88). Critically, treatment strategies provide a practical starting point for exploring disinfection opportunities, as manure processing may indirectly also have benefits for reducing infectious virus in the manure (aerobic or anaerobic digestion, addition of chemical treatments, etc.) and will be discussed further in Section 4.

#### 5.1.4 Manure application

Manure application is typically seasonal, as climate conditions (heavy rains, intense heat, freezing) can impede effective application. In summer, warm temperatures promote evaporation, odor dispersal, and loss of nutrients, whereas in winter, frozen ground contributes to manure runoff and eutrophication (76). Soil saturation levels and crop nutrient management plans can also affect application schedules (89). The method selected to apply manure is based on infrastructure, local regulations, and the physical characteristics of the manure. To apply liquid manure, it may need to be resuspended with pumps or agitators to incorporate solids which have sedimented to the bottom of the lagoon (70). Liquid manure is transported to the application site with irrigation, tanker, or tractor-mounted systems and distributed through broadcasting, dribble banding, or injection techniques, whereas solid manure is distributed onto fields via box or hopper spreaders (70, 78, 89). Proper application optimizes manure's nutrient potential and minimizes nitrogen leaching. The cumulative steps taken throughout manure management are critical in ensuring that manure is transported from holding facilities and distributed across acres of land are safe and free of pathogenic contamination. From a biosecurity perspective, the application stage represents a key point in which potentially hazardous materials come into contact with largely exposed terrain (90). In a successful manure management model, pathogens and toxins present in raw manure will be sufficiently accounted for and reduced, to ensure safe crop production and minimal environmental contamination.

### 5.2 Manure management in developing systems

When compared to the strategies described above, agricultural systems found in developing regions often lack substantive investments for infrastructure, critically shaping accepted farming practices. Additionally, it is important to note that while certain countries are considered as either developed or developing by the United Nations, there is heterogeneity within countries (91). Farms which rely on more modest, traditional methods can be found around the globe, regardless of national economic standing. While Canada and the United States are considered developed countries, strict biosecurity should not be assumed nor taken for granted, as smaller farm management approaches may align more closely with a developing system. Canada's pork industry is dominated by large-scale commercial operations, however there are numerous smaller producers, often family-owned or run on a small scale, ranging from a few pigs to larger hobby operations. These farms, particularly in rural areas, experienced a surge in popularity during the COVID-19 pandemic. While they have fewer animals and less structure compared to large animal operations, these farms are still expected to meet quality assurance programs to ensure animal welfare and traceability (92).

Teenstra et al. (63) reviewed barriers across the globe which hinder successfully integrated manure management, highlighting lack of knowledge/infrastructure, ineffective policies, and insufficient investment as key factors. When considering collection strategies, methods are often adopted which require lower infrastructure investment. In Asia, solid manure is often scraped or shoveled from floors and kept in piles (78). Collected manure is applied to crops or incorporated as feed for fish farms, which, while resourceful, can compromise biosecurity and expand disease transmission. In Africa, smallholder farms also typically collect solid manure. Larger farms may utilize liquid manure systems, though these are less common due to economic constraints (93). Ibrahim et al. (94) surveyed Ugandan pig farmers and found that 46% of farmers store and handle urine separately from feces to reduce odor. Of these, 45% discharge urine directly into the environment. Ewuziem et al. (95) reported dumping and incineration are common methods of disposal in Nigeria, in which farmers dump wastes into spaces on- or off-farm. Manure is heaped into large piles and set on fire during the dry season. The strategy, while widely accepted and will kill pathogens, is hazardous for health and creates environmental and odor concerns. Other methods included using manure for crops or fish farming, or dumping into streams, leading to contamination concerns as previously mentioned. Ewuziem et al. (95) concluded that more efficient management methods are necessary to alleviate drawbacks of these methods and promote sustainable pig farming. Similarly, in Dominican Republic (a region with a recent ASF outbreak), swine manure is often directly discharged into rivers (96) due to a lack of investment in infrastructure. Minimal infrastructure is common amongst other countries in Latin America, where roughly 80% of farms are smallholder or family farms (86).

Due to lack of resources, most developing manure management systems lack full integration (63). Manure is often improperly stored and discharged into the environment including rivers, ponds and lakes, representing a threat to ecological health and weakening biosecurity. This aspect of manure management highlights a critical concern for the ongoing spread of high consequence animal diseases, such as ASF. Until a safe and effective ASF vaccine is developed, on-farm biosecurity measures are the best strategy to preserving herd health. However, strict biosecurity is contingent on having the economic resources to support the necessary equipment, personnel, and infrastructure. Well-designed manure management strategies are integral to upholding biosecurity, and in developing regions with less established operations, ineffective managing (ie: improper cleaning and disinfection, dumping waste, manure piles within reach of wild pig populations, etc.) can promote spread of disease (97–99). In an article comparing highly pathogenic avian influenza (HPAI) and ASF surveillance and fatality metrics, Kim et al. (100) reported that nearly 75% of ASF outbreaks reported between January 2016 and December 2023 occurred on backyard farms, compared to 13.5% on commercial farms, and 11.6% in villages, highlighting these premises as susceptible to ASFV outbreaks. Economic supports to encourage better management practices, along with frequent surveillance, can assist backyard and smallholder farmers to minimize risk for ASF, identify any cases quickly, and to limit the chance of farm-to-farm transmission.

## 6 Manure management during ASF outbreaks

As demonstrated throughout the previous sections, integrated manure management is an innately complex series of processes. The challenges of correctly managing such an intricate system are heightened still in the event of an animal disease outbreak. Since ASFV is shed in feces and urine from the onset of symptoms (16), all forms of manure are potentially infectious. During an outbreak, manure must be disinfected and disposed of in a way that upholds biosecurity, while considering feasibility, costs, environmental impact, and potential hazards. To account for the diversity of farming operations across Canada, various methods of manure treatment and disinfection should be considered to meet the unique needs of each farm. Some methods, such as chemical disinfection, are currently accepted as treatment strategies in regions which have experienced ASF outbreaks (ie: Spain, Vietnam, etc.) (101, 102). Other methods, such as ultraviolet (UV) treatment, have yet to have their efficacy and applicability proven for treatment of ASFV (62). This section reviews potential lines of action and considers how they apply to the North American context.

### 6.1 Fallowing

Allowing infectious manure to fallow for extended periods may be effective in inactivating virus. However, various factors affect the persistence of virus in manure, including pH, temperature, soil composition, organic material, physical condition of the virus, ammonia concentrations, UV exposure, and vestigial effects of detergents and sanitizers used inside the hog barns (62, 103). Of particular relevance to Canadian climates, colder temperatures are known to promote virus survival (29, 104, 105). Porcine epidemic diarrhea virus (PEDV) has been observed to survive in manure storage for up to 9 months in Canadian climates, with seasonal temperatures ranging from −30 °C to 23 °C (106). However, despite the protective effects of cold temperature on virus stability, there is little evidence whether this contributes to increases in outbreaks on regions currently experiencing ASF outbreaks. Incidence of ASF infection in domestic pigs is typically associated with summer/autumn, when animal and human movement are higher and lead to farm-to-farm transmission (107, 108). During winter months, it is less likely that contaminated manure will contribute to disease incidence, as manure application is limited to spring and autumn months. Although ASFV contaminated lagoons may pose less immediate risks during colder seasons, they likely serve as a strong reservoir for viable virus, which may contribute to indirect transmission when manure is spread and human movement around lagoons is more frequent. It is critical that virus stability and infectivity is studied to detail how seasonal perturbations influence disease risk. However, owing to the inherent unpredictability of climate observed year-to-year, it is difficult to predict the precise point of virus inactivation.

As previously mentioned, several studies have shown the shedding of ASFV in pig and boar excretions. Davies et al. (16) showed survival of infectious ASF virus in pig feces up to 5 days at 4 and 12 °C, 3 days at 21 °C and 1 day at 37 °C. In urine, virus survived up to 5 days at 4, 12, and 21 °C and 1 day at 37 °C. The ASFV genomic material was detected in feces up to 98 days at 4 and 12 °C, 35 days at 21 and 37 °C and in urine for up to 126 days at all temperatures. Based on the half-life of ASFV in these matrices, feces and urine could remain infectious for 8.5 and 15.3 days at 4 °C, and 3.7 and 2.9 days at 37 °C, respectively. Fischer et al. (109) detected ASFV genomic material in urine and feces for up to 6 months at −20 °C, 3 months at 4 °C, and 1 week at room temperature. However, they detected no infectious virus in urine or feces after a week, regardless of storage temperature. Similarly de Carvalho Ferreira et al. (17) determined the viral DNA in wild boar feces stored at 4 °C and 12 °C to be 4 and 2 years respectively, largely protected by colder temperatures. In warmer temperatures, ASFV DNA half-life was approximately 22 days at 20 °C and 15 days at 30 °C. Kosowska et al. (18) had similar findings in wild boar infected with ASFV, with virus being detected in feces in groups infected with either attenuated or virulent strains of ASF.

The properties of the soil, including the pH, structure and ambient temperature can vary in earthen lagoons across regions, and may affect the fallowing process (110). Furthermore, barn surfaces can influence virus survival, with virus remaining viable in contaminated pig pens for up to 1 month, depending on the surface type (13, 16). Haas et al. (105) proposed between 60 and 180 days as a reasonable safety margin for ASFV to be inactivated depending on the manure storage condition. The European Union has recommended that slurry should be stored for at least 60 days and that solid manure should be stacked, sprayed with disinfectant, and allowed to sit for 42 days before incineration or burial (111). Ultimately, ASFV survival in a pit or lagoon is uncertain and likely varies across farms. Research is needed to determine safe treatment to inactivate virus before slurry can be approved for transport or land application.

While more work is needed the evaluate the effectiveness of long-term lagoon storage to inactivate ASFV, the practice is simple in design, limits the potential for cross-contamination during more complicated processing, has minimal resource inputs and environmental repercussions, and requires less skilled labor compared to other approaches (62). However, the farm cannot repopulate while the lagoon is left to fallow, resulting in prolonged loss of revenue for the farmers. Additionally, in the event of heavy precipitation events, lagoons may exceed capacity and require pumping into alternative storage, increasing costs and spreading of ASFV contaminated material. More research is necessary to understand how physical, biological, and chemical factors of lagoons affect reduction of ASFV during fallowing.

### 6.2 Chemical decontamination

Chemical disinfection is widely employed for decontamination of surfaces and materials which have contacted ASF positive animals (13). Studies have shown the efficacy of chemicals to reduce infectious particles under diverse experimental conditions (Table 3). Juszkiewicz et al. (20) reported the effectiveness of sodium hypochlorite, acetic acid, phenol, glutaraldehyde, caustic soda, and potassium peroxymonosulfate as viable disinfectants against ASFV in solution. Krug et al. (28) studied disinfection of pork processing plant surfaces (namely concrete, steel, and plastic surfaces) and found quaternary ammonium compounds and sodium hypochlorite to be effective under certain conditions, whereas citric acid and sodium hypochlorite had reduced efficacy when in the presence of organic material (ie: blood). Broadly, organic material greatly interferes with the virucidal activity of many reagents, and in particular, the dense organic content of manure presents a uniquely challenging medium to treat (20).

**Table 3:** Compounds identified as potential disinfectants for African swine fever virus.

Disinfectant	Concentration	Conditions of application (temperature and/or time)	Outcome	References
Calcium hydroxide (hydrated lime)	0.5%−1%	30 mins at 4 °C and 22 °C (manure 2.3% solid content)	Inactivated	(24, 120)
	0.5%	2.5–5 mins at 4 °C (manure 2.3% solid content)	Inactivation below detectable level	(24)
Sodium carbonate (washing soda)	4%−10%	10–30 mins (unspecified contact temperature)	Inactivated	(149)
Sodium hydroxide (caustic soda)	8/1,000	30 min (unspecified contact temperature)	Inactivated	(120)
	0.5%−1%	30 mins at 4 °C and 22 °C (manure 2.3% solid content)	Inactivated	(24)
	1%	2.5–5 mins at 4 °C (manure 2.3% solid content)	Inactivation below detectable level	(24)
	2%	30 mins at 10 °C	>4log_10_ reduction	(20, 150)
Sulfuric acid	1%	15 min to 1 week	Inactivated	(115)
Formic acid	1:800	30 mins at 20 °C	>4log_10_ reduction	(151)
	1%−4%	15 mins to 1 week	Inactivated	(115)
Formaldehyde	1%	15 mins (unspecified contact temperature)	Inactivated	(115)
Sodium dodecyl sulfate	3%	15 min (unspecified contact time)	Inactivated	(115)
Glutaraldehyde	0.1%−1%	30 mins at 10 °C	>4log_10_ reduction	(20)
Sodium hypochlorite	Between 0.03% and 0.5% chlorine	30 mins (unspecified contact temperature)	Inactivated	(120)
	0.03%−0.0075%	30 mins at room temperature	Inactivated	(150, 152)
	1,000–2,000 ppm	30 mins at 22 °C	>4log_10_ reduction (below detection limit)	(27, 153)
	Between 1% and 1.5%	30 mins at 10 °C	>4log_10_ reduction	(20)
Ortho-phenylphenol	3%	30 mins (unspecified contact temperature)	Inactivated	(120)
Formalin	3/1,000	30 mins (unspecified contact temperature)	Inactivated	(120)
Peroxide	20.6–102.9 mM H_2_O_2_	10–20 mins at 48 °C	>4log_10_ reduction (below detection limit)	(154)
	1.73 mg/liter (1,211 ppm)	30 mins at 20–40 °C	< 10 TCID_50_ (50% Tissue culture infective dose)	(155)
Citric acid	1%−2%	10–30 mins at 22 °C	>4log_10_ reduction (below detection limit)	(27, 28)
	1%	2 h at 10 °C	2–3 log_10_ reduction	(156)
Acetic acid	2%−3%	30 mins at 10 °C	>4log_10_ reduction	(20)
Peracetic acid	3%	15 mins (unspecified contact temperature)	Inactivated	(115)
	0.1%	2 h at 10 °C	2–4log_10_ reduction	(156)
Phenol compounds	1%	30 mins at 10 °C	>4log_10_ reduction	(20)
Potassium peroxymonosulfate	0.5%−1%	30 mins at 10 °C	>4log_10_ reduction	(20)
	2%	10 mins at room temperature	Reduced to limit of detection	(28)
	1:200	5 mins at 4 °C/30 mins at 20 °C	≥3log_10_ reduction	(114)
Iodine compounds/Iodophor (iodine 1-3%)	1:750	30 mins at 20 °C	>4log_10_ reduction	(151)
	0.015%−0.0075%	30 mins at room temperature	Inactivated	(152)
	1%−5%	5–30 mins at room temperature	>5log_10_ reduction	(157)
Quaternary ammonium compound	1:3,200 (0.003%)	30 mins at room temperature	Inactivated	(152)
	1:200-1:400	5 mins at 4 or 20 °C	≥3log_10_ reduction	(114)
	800 ppm in 400 μl volume	5–10 mins at room temperature	3–4log_10_ reduction	(28)
	1%	30 mins at 10 °C	≥4log_10_ reduction	(20)
Acidified electrolyzed water	40–140 ppm free chlorine	30 mins at 4 °C	≥4log_10_ reduction	(158)
Ozonized water	10–20 mg/L	3-10 mins at room temperature	≥3log_10_ reduction	(159)
Virkon^TM^ S	2%−5%	10 mins (unspecified contact temperature)	Inactivated	(113)
	1%	30 secs at 37 °C	≥5.5log_10_ reduction	(160)
One-Stroke Environ (o-Phenylphenol (10%), o-Benzyl-p-chlorophenol (8.5%), p-tert-Amylphenol (2%)	0.5%−1%	30 mins (unspecified contact temperature)	Inactivated	(161)

Disinfectant groups suggested for application to ASFV include acids, alkalis, aldehydes, chlorine and chlorine compounds, iodine compounds, oxidizing agents, phenolic compounds, and quaternary ammonium compounds (62, 112). Turner and Williams (24) evidenced effective decontamination of ASF infected pig slurry using sodium and calcium hydroxide applied in a 0.5% (w/v) solution to inactivate virus after 150 s at 22 °C. Furthermore, 1.0% (w/v) preparations of the solutions were able to inactivate the virus for the same exposure time at 4 °C. Turner and Burton (23) additionally suggested ozonation, chlorination, ethylene oxide treatment, paracetic acid, iodine detergent, and ammonia as potential treatments for the inactivation of viruses in pig slurries. Gabbert et al. (113) demonstrated ASFV disinfection on concrete and stainless-steel surfaces with Virkon, a peroxymonosulfate compound certified by the USDA for disinfection in the event of an outbreak. The efficacy of peroxymonosulfate based compounds was further supported by Sovijit et al. (114), who also tested quaternary ammonium compounds, and found both to be effective in reducing ASFV particles. Currently, recommendations by The Global African Swine Fever Research Alliance suggest that ASF contaminated liquid manure can be treated for a week with 1% formaldehyde, 4% formic acid, 2% sodium dodecyl sulfate, or 1% glutaraldehyde to inactivate the virus (115). Additionally, the USDA recommends that chemical disinfectant application for foreign animal disease should alter the pH to either < 2 or >11 while stirring for at least 7 days, as ASFV is stable over a pH range of 4 to 10 (116, 117).

Alongside their efficacy, however, chemical compounds also carry considerable drawbacks. Inorganic acids are typically avoided for large-scale decontamination, as they are corrosive in nature. Additionally, organic acids and sodium hypochlorite have reduced effectiveness in materials with high organic content, such as manure (62). Aldehydes are temperature dependent, and furthermore, many of the chemicals with strong virucidal activity are also toxic to humans and the environment. High levels of ammonia in manure further complicates the disinfection process, as addition of alkalis to lower the pH of manure can result in the formation of various harmful gasses (118). Moreover, manipulating the pH of the lagoon toward extreme acidity or alkalinity requires neutralization, generating hazardous waste products (116).

Chemical disinfection carries significant occupational and environmental hazards which require consideration before their widespread adoption. Environmentally friendly disinfectants are being explored, such as plant extracts or acidifying powders (26, 119), however, these solutions will need to be explored to assess their applicability for large-scale disinfection efforts. Chemical treatment of agricultural lagoons may be impractical due to the scale, technical logistics, and health hazards associated with harsh chemicals (62). However, in the event of an ASF outbreak, all options should be considered and may prove beneficial for certain circumstances. Further study is necessary to demonstrate efficacy, practicality, and how prescribed treatments interact with the innate chemistry of manure.

### 6.3 Thermal decontamination

The application of heat to stored manure could be a potential approach to inactivating ASFV on swine farms during ASF disease outbreak, although the technical feasibility of this approach has yet to be demonstrated. Turner and Williams (24) investigated the viability of heat treatment in their work in the late 1990′s, and demonstrated that ASFV was not detectable after 30 min of heat treatment between 50 °C and 60 °C. ASFV in slurry was inactivated after only 2–5 min at 56 °C and within 2–3 min at 60 °C (depending on its total solid content), and at 65 °C, all virus was inactivated within 60 s, regardless of solid content. Haas et al. (105) had previously identified pasteurization between 65 °C and 100 °C for 30 min and microwave treatment as effective against ASFV. As previously mentioned, a similar study by Botner and Belsham (37) evidenced heat treatment was able to reduce other animal viruses, but found DNA viruses to be more resistant to heat compared to RNA viruses. Thus, it is important to appreciate the physical resiliency of ASFV when applying heat treatment.

Broadly, the WOAH recommends heat treatment for inactivation of ASFV for 70 min at 56 °C or 20 min at 60 °C and specifically recommends that manure is treated at a minimum of 55 °C for 60 min, or at 70 °C for at least 30 min (14, 120). However, the experiments conducted to support these recommendations in slurry was conducted several decades ago (24, 105). Novel research is needed to reliably demonstrate the stability of ASF in swine slurry over various temperatures and to verify inactivation infectious virus through thermal treatments.

While a heat treatment approach avoids the introduction of harsh chemicals and can efficiently decontaminate manure slurry, implementing heat treatment to entire pits or lagoons may not be feasible. Unless specialized pasteurization equipment is previously installed on a farm, it is unlikely that purchasing and implementing such equipment is practical in the event of an outbreak. Moreover, the energy and mechanical equipment required to physically process the high volumes of manure slurry that require disinfection may serve as a barrier for many farming operations.

### 6.4 Anaerobic digestion

As previously described in Section 5.1.3, anaerobic digestion is a manure treatment strategy that is gaining traction globally for production of valuable biofuel and ability to reduce methane emissions (84). While the approach requires capital investment, the process partially offsets the initial costs by providing a renewable energy source, which can be increasingly important in regions which experience energy scarcity. Anaerobic digestion can either occur in moderate (30 °C−37 °C) or high (50 °C−60 °C) temperatures ranges (62). As previously stated, ASFV is susceptible to high temperatures, and thermophilic digestion should therefore inactivate viral particles. However, this theory needs to be experimentally proven (121). Moreover, whether lower temperature digestive methods can also inactivate virus should also be explored. While the strategy of anaerobic digestion provides an eco-friendly alternative to conventional manure treatment methods, the technology has yet to be widely adopted in Canada and the United States (85), and therefore, its usefulness in treating ASF outbreaks will be limited until it becomes more readily employed.

### 6.5 Alternative approaches

In addition to the more conventional treatment options previously described, a number of experimental strategies have been mentioned in literature which may provide benefits for certain situations, including UV treatment, ozonation, cavitation, photocatalytic inactivation, gamma and electron beam radiation, and chlorination (23, 62), however, many of the strategies are only applicable to clarified liquids and rely on initial separation of solid and liquid fractions. Most manure slurry or liquids have high turbidity and are unlikely to be responsive to such treatments. Other methods, such as irradiating manure, are costly and carry significant safety concerns, thus reducing their appeal as implementable solutions. Moreover, while aerobic treatment has been shown to reduce other viruses present in swine manure (62, 122), no experimental studies have been conducted to consider this approach for reduction of ASFV in contaminated manures. Interestingly, composting was shown to be an effective strategy to reduce ASFV in contaminated boar carcasses (58), suggesting the treatment option should be investigated in future efforts. Moreover, composting has been considered as an effective strategy for inactivating FMDV in cattle manure during a potential outbreak, as aerobic composting is capable of reaching 60 °C, enabling thermal inactivation of the virus particles (123). However, with most hog operations in North America storing manure in deep pits or lagoons void of oxygen, incorporating solid liquid separation and aerobic digestive treatments for large volumes on these operations is unlikely to take place (62). Additionally, FMDV is more susceptible to pH compared to ASFV, so comparing these disease treatment outbreaks should be done with caution (124).

## 7 Discussion and future perspectives

Within Canada, swine farming operations typically collect slurry beneath slatted floors, and pump the manure mixture to a lagoon, before application to local crop fields or transportation to other farms. This approach to manure management is advantageous not only for its simplicity, but also its ability to amend the soil of vast areas of farmland. The success of Canada's pork producing sector and other agricultural commodities are critically tied to well-executed manure management practices, which promote herd, crop, and soil health. In the event of an ASF outbreak, manure disinfection strategies will need to align with the currently adopted manure management approaches to facilitate rapid implementation. Strategies which employ pre-existing infrastructure, such as holding ponds or lagoons, are the most practical solutions in outbreak scenarios. Treatments which require additional economic investment when resources are already strained, or skilled labor by staff who are tasked with the significant pressures of an animal disease outbreak are impractical and may be less effective in stemming the spread.

Overall, simple methods, such as fallowing, chemical disinfection, and heat treatment, are the most promising strategies which would be easy to implement rapidly in the event of ASF detection. Additionally, synergistic interactions between such treatments (i.e., adjusting slurry toward an alkaline pH during exposure to moderately elevated temperatures) may be effective in reducing infectious virus and warrant consideration and research. Allowing manure lagoons to fallow is the most straightforward method; it requires no additional resource inputs and if strict containment is maintained, represents a relatively low risk for disease transmission. Moreover, it is the simplest method to apply for large volumes of contaminated manure. In a study assessing the survival of PEDV in manure storage in the Canadian province of Manitoba, it was noted that environmental temperatures, UV light and sunlight, and pH were influential in virus stability within earthen manure storage lagoons (106). In particular, Canada experiences prolonged winter seasons with extremely cold temperatures regularly below freezing. It can be expected that such external factors would largely affect the survival of ASFV on Canadian swine farms and their role should be investigated in experimental study. Moreover, authors of the same study found that PED viral load varied between different stratified layers within the storage lagoon, with the prevalence of viral genomic material throughout the layers changing over time. The authors hypothesized that PEDV detection may initially be high in the liquid fraction, but as the slurry settles, virus particles will migrate into the sludge fraction. Virus detection was lower in the liquid fraction of the lagoon at later dates, likely due to exposure to the sunlight. The way ASFV precipitates in the manure matrix over time, as well as its interactions with various strata of solid contents in the lagoon will also need to be explored.

Chemical and thermal disinfection are also promising solutions, depending on the volume and location of manure that needs to be treated. Application of chemical disinfectants may be more practical when treating smaller batches of contaminated waste or in contained settings with little risk of possibility for chemical run-off and environmental hazard. Similarly, bringing entire basins of stored manure up to the WOAH recommended temperatures of more than 55 °C, while maintaining the temperature uniformly and consistently for extended periods, may prove impractical. While less optimal for large-scale batch treatment, chemical and thermal disinfection may be useful within certain decontamination situations, such as treatment of manure within barns, transport trucks, or pump wells.

After reviewing the current literature pertaining to ASFV disinfection in swine manure, it is apparent that the research area still requires extensive study. Specifically, the survivability and infectivity dynamics of ASFV in slurry during prolonged storage, or after chemical or thermal disinfection, needs to be fully elucidated to inform effective manure management solutions. Since ASF has not entered Canada, study of the virus is strictly confined to laboratory settings. Field study of ASFV in real manure lagoons is not possible, and therefore, laboratory-based study, while limited, serves as the best option to inform effective treatment measures. However, in addition to being restricted to biosafety level 3 laboratories, studying virus in slurry is innately complex. Slurry is often toxic to cell culture, houses a highly complex and variable microbiome, and readily absorbs virus, leading to challenges isolating the virus. While current recommendations are helpful in guiding regions currently experiencing ASF outbreaks, extensive experimental study is needed to accurately verify the reduction of infectious virus under realistic conditions.

In particular, the environmental conditions experienced by pig farmers across Canada are highly variable. Livestock herd size, precipitation patterns, soil chemistry, bedrock, and seasonal temperatures of farms across such a large geographic region reflect immense diversity, presenting many factors which must be appreciated when recommending effective courses for treatment. The intrinsic characteristics of slurry are also highly heterogenous. Across farm operations, slurries will range in solid content levels, chemical parameters, microbiota, and treatment methods. Future study must investigate how slurries from different regions are affected by Canadian climatic factors and soil types. ASF outbreak strategies should consider the multifaceted nature of the task at hand. Additionally, the economical, occupational, and environmental ramifications of recommended treatments need to be reviewed and assessed to ensure that treatments are safe in all aspects and arise from a comprehensive One Health approach. Research efforts to prepare for potential ASF introduction into Canada should reflect the Canadian pork sector's unique characteristics, formidable scale, and the critical role it plays in the nation's economy and food security.

To our knowledge, this narrative review is the first to review relevant literature and describe the gap in manure management strategies for ASFV affected farms. Despite their integral role in modern agricultural systems, manure management practices are rarely discussed in veterinary science research, and this paper provides readers with a working understanding of important concepts to appreciate the complexity and diversity of these systems. This review integrated key concepts of multiple disciplines to encourage scientific discussion and collaboration to explore and advance a research topic which has been neglected by focusing study on virology or agricultural sciences alone. Additionally, since ASF has not been detected in the North American continent, our review ensures ASF preparedness remains salient among those involved in animal research, despite other animal disease, such as highly pathogenic avian influenza, receiving more attention in recent years (125–128). However, the review was limited by the lack of availability of recent literature to inform precise methods to treat manure pits. It was clear early in the review process that strategies relied upon to disinfect contaminated manure during ASF outbreaks are not well reported in academic, peer-review literature. Recommendations from animal health organizations are an important part of the conversation, but data to support their recommendations are often not verified by experimental data in the field, or may be difficult to rapidly implement during an outbreak (14). There is a marked deficiency of published literature, particularly peer-reviewed study with robust experimental design, to support meaningful strides toward a definitive treatment strategy that incorporates practicality, availability of financial resources, and environmental health. While a narrative review is beneficial for beginning the discussion on ASF manure management, systematic reviews should be conducted in the future when more research is published in this field. Ultimately, this work aims to identify this critical gap in literature and challenge the scientific community for future exploration.

## 8 Conclusion

Manure management is an essential component of biosecurity and disease control in the event of an ASF outbreak, especially given its role as a potential transmission pathway for the virus and the severe impacts on the pork industry and the Canadian economy. This review highlights the different manure management practices across various continents and discusses existing guidelines for treatment and disinfection during ASF outbreaks. However, significant gaps remain, including limited literature and research on manure management strategies during ASF outbreak, insufficient empirical evidence on the effectiveness of various treatment methods, and inadequate integration of manure management within broader biosecurity plans. Given the risk of ASF transmission through manure, further research is essential to address these gaps and develop robust, context-specific protocols for managing manure during ASF outbreaks. Coordinating high-quality scientific experiments and proactively organizing strategic response efforts will bolster the pork industry's resiliency and promote prosperity for the Canadian agricultural sector in the years to come.
